# Propensity scores as a novel method to guide sample allocation and minimize batch effects during the design of high throughput experiments

**DOI:** 10.1186/s12859-023-05202-6

**Published:** 2023-03-07

**Authors:** Patrick M. Carry, Tim Vigers, Lauren A. Vanderlinden, Carson Keeter, Fran Dong, Teresa Buckner, Elizabeth Litkowski, Ivana Yang, Jill M. Norris, Katerina Kechris

**Affiliations:** 1 Colorado Program for Musculoskeletal Research, Department of Orthopedics, University of Colorado Anschutz Medical Campus, 12631 E. 17Th Ave, Room 4602, Mail Stop B202, Aurora, CO 80045 USA; 2 Department of Epidemiology, Colorado School of Public Health, Aurora, CO USA; 3 Department of Biostatistics and Informatics, Colorado School of Public Health, Aurora, CO USA; 4 Barbara Davis Center for Diabetes, University of Colorado Anschutz Medical Campus, Aurora, CO USA; 5 Department of Medicine, University of Colorado Anschutz Medical Campus, Aurora, CO USA

**Keywords:** Batch effects, Propensity scores, Batch effect adjustment, ComBat

## Abstract

**Background:**

We developed a novel approach to minimize batch effects when assigning samples to batches. Our algorithm selects a batch allocation, among all possible ways of assigning samples to batches, that minimizes differences in average propensity score between batches. This strategy was compared to randomization and stratified randomization in a case–control study (30 per group) with a covariate (case vs control, represented as β1, set to be null) and two biologically relevant confounding variables (age, represented as β2, and hemoglobin A1c (HbA1c), represented as β3). Gene expression values were obtained from a publicly available dataset of expression data obtained from pancreas islet cells. Batch effects were simulated as twice the median biological variation across the gene expression dataset and were added to the publicly available dataset to simulate a batch effect condition. Bias was calculated as the absolute difference between observed betas under the batch allocation strategies and the true beta (no batch effects). Bias was also evaluated after adjustment for batch effects using ComBat as well as a linear regression model. In order to understand performance of our optimal allocation strategy under the alternative hypothesis, we also evaluated bias at a single gene associated with both age and HbA1c levels in the ‘true’ dataset (CAPN13 gene).

**Results:**

Pre-batch correction, under the null hypothesis (β1), maximum absolute bias and root mean square (RMS) of maximum absolute bias, were minimized using the optimal allocation strategy. Under the alternative hypothesis (β2 and β3 for the CAPN13 gene), maximum absolute bias and RMS of maximum absolute bias were also consistently lower using the optimal allocation strategy. ComBat and the regression batch adjustment methods performed well as the bias estimates moved towards the true values in all conditions under both the null and alternative hypotheses. Although the differences between methods were less pronounced following batch correction, estimates of bias (average and RMS) were consistently lower using the optimal allocation strategy under both the null and alternative hypotheses.

**Conclusions:**

Our algorithm provides an extremely flexible and effective method for assigning samples to batches by exploiting knowledge of covariates prior to sample allocation.

**Supplementary Information:**

The online version contains supplementary material available at 10.1186/s12859-023-05202-6.

## Background

Batch effects represent non-biological sources of variation in high throughput experiments [1–3]. Batch effects are often associated with a greater magnitude of differential expression than the primary biological covariate(s) of interest [2]. Correlation between these technical factors and biological effects has the potential to mask true associations or lead to spurious findings [2, 4–6]. Leek et al. [2] identified batch effects in nine large, publicly available datasets. Moreover, up to 99.5% of the features in the datasets were significantly correlated with one or more of the technical variables within the individual datasets [2]. Batch effect concerns extend beyond the individual studies. Failure to account for batch effects can confound meta-analyses and decrease reproducibility of study results leading to an inefficient use of resources [2, 4].

Following data acquisition, numerous batch correction methods exist, ranging from simple options including explicitly modeling known technical factors in a multivariable analytic framework to more complex algorithms [1, 3, 5]. Surrogate variable analysis (sva) is a popular method in which, consistent with other approaches, batch effects are estimated from the data [7], capturing both known and unknown technical factors. The factors estimated using this data-driven approach are then corrected for in subsequent analyses [7]. Removing unwanted sources of variation is another method that is conceptually similar to the sva approach except that it exploits biological features assumed to be independent of the covariate of interest, colloquially termed ‘housekeeping genes’, to remove batch effects [8]. Known batch effects can be specified and removed using a parametric or a non-parametric empirical Bayes approach [9]. This popular method is often implemented using the ComBat function within the sva r package[7]. Less commonly used batch correction methods include the machine learning based Distance-weighted discrimination method [10], mean centering [11], geometric and arithmetic ratio methods [12], and location/scale adjustment models [13]. However, even when the experimental outcome (i.e. case in a case–control study) is equally distributed across batches, imbalance in the distribution of biologically relevant covariates across batches has the potential to complicate batch correction methods [14] as it is challenging to differentiate between confounding effects and true biological effects. Therefore, sound experimental designs using methods that minimize imbalance in covariates across batches should be prioritized.

Propensity scores are used in observational studies to address imbalance in important covariates across levels of the exposure/treatment variable. In the context of a treatment study (active treatment vs control), propensity scores represent the probability of receiving active treatment conditional upon a set of covariates [15]. Within the causal inference framework, propensity scores are used to address the ‘exchangeability assumption’ or the assumption that the potential outcomes are independent of the treatment assignment and thus, the treated group would have developed the same average outcome as the untreated group had they not been assigned to the treatment group (we refer the reader to Hernan and Robins [16] for more additional information about the causal inference framework). In this way, propensity score matching, stratification and/or weighting are used to ensure the probability of being assigned to the treatment group given a complete set of clinically relevant covariates is balanced between groups [16–20], potentially satisfying one of many assumptions required for estimation of causal treatment effects. Propensity scores are also useful as a dimension reduction technique, providing a single value, ranging between 0 and 1, that represents the overall balance in the distribution of a set of covariates between groups. In the context of batch assignments, differences in propensity scores between batches can be used to screen for imbalance in the overall distribution of a set of covariates across two or more batches. Therefore, we propose using propensity scores to guide allocation of samples to batches in order to identify the optimal allocation iteration that minimizes the differences in propensity scores between the batches. In this way, we build on the methods for implementing covariate constrained randomization in cluster randomized controlled trials [21] to provide a more optimal methodology for allocating samples to batches during the design of high throughput experiments.

## Results

### Study population

In order to create a biologically plausible scenario, we downloaded a publicly available microarray gene expression dataset (GSE50397), that includes gene expression data from human pancreas islet cells. Biologically relevant variables in the phenotype file (age and HbA1c) were used to represent potential confounding variables that differ between cases and controls in a typical case–control design. Consistent with the experimental design, there was a significant difference in both age (49.3 yrs. ± 10.9 vs. 62.9 yrs. ± 6.2, *p* < 0.0001) and HbA1c (5.2 ± 0.4 vs. 5.8 ± 0.3, *p* < 0.0001) levels in cases (*n* = 30) versus controls (*n* = 30).

### Bias prior to batch correction

**Fig. 1:**
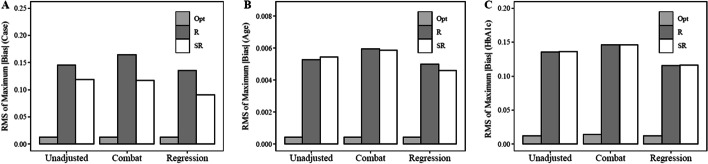
Root mean square (RMS) of maximum absolute bias across the experimental scenarios. Within each experimental iteration, absolute bias was calculated as the absolute value of the difference between observed beta coefficient and the ‘true’ coefficient across the 10,000 most variable genes in the expression dataset. Maximum absolute bias represents the maximum absolute difference between the true beta (prior to adding batch effects) and the beta estimates from each of the experimental scenarios (Table 1). The root mean square or standard deviation of the maximum absolute bias estimates across the experimental simulations was estimated for ${\upbeta }_{1}$ (panel **A**, case vs control parameter that was set at 0 to represent the null hypothesis), ${\upbeta }_{2}$ (panel **B**, parameter estimate representing the association between age and gene expression), and ${\upbeta }_{3}$ (panel C, parameter estimate representing the association between HbA1c and gene expression. Opt = optimal allocation strategy, R = simple randomization strategy, SR = stratified randomization strategy

Under each experimental scenario (Table 1), we estimated bias as the difference between each of the beta values (${\upbeta }_{1}$, cases and controls, ${\upbeta }_{2}$, increase in expression for every 1 unit increase in age, and ${\upbeta }_{3}$, increase in expression for every 1 unit increase in HbA1c levels) and the ‘true’ beta values. The ‘true’ beta values were calculated using gene expression values from GSE50397 before the simulated batch effects were added to the expression matrix. The beta value for the case variable, ${\upbeta }_{1}$, was set at zero and thus represents the effectiveness of the batch allocation strategies under the null hypothesis, no difference between cases and controls. Prior to batch correction, (no adjustment), average maximum absolute bias was lowest, closest to the true ${\upbeta }_{1}$ values, in the optimal condition (Table 2). In many cases, bias values achieved using the optimal allocation strategy were closest to the ‘true’ values by an order of magnitude relative to the other approaches (Table 2). Variability in maximum absolute bias as represented by RMS of maximum absolute bias for ${\upbeta }_{1}$ was also lowest using the optimal condition Table 2 and Fig. 1). This finding was also consistent across ${\upbeta }_{2}$ (age) and ${\upbeta }_{3}$ (HbA1c).

**Table 1:** Summary of experimental scenarios

Batch allocation strategy	Adjustment method		
Optimal	No adjustment	ComBat adjustment	Regression adjustment
Randomization	No adjustment	ComBat adjustment	Regression adjustment
Stratified randomization	No adjustment	ComBat adjustment	Regression adjustment

**Table 2:** Average maximum absolute bias or difference between true* and batch effect conditions prior to batch adjustment

		Average max	RMS
Case (${\upbeta }_{1})$	Randomization versus True	3.06E-01	1.45E-01
	Stratified Randomization versus True	2.81E-01	1.18E-01
	Optimal versus True	**1.72E-01**	**1.25E-02**
Age (${\upbeta }_{2})$	Randomization versus True	1.12E-02	5.29E-03
	Stratified Randomization versus True	1.12E-02	5.45E-03
	Optimal versus True	**6.18E-03**	**4.28E-04**
$HbA1c ({\beta }_{3})$	Randomization versus True	2.96E-01	1.35E-01
	Stratified Randomization versus True	2.95E-01	1.36E-01
	Optimal versus True	**1.65E-01**	**1.21E-02**

True* condition represents gene expression values from GSE50397 before batch effects were added to the expression sets in each of the 1000 simulation iterations

Within each experimental iteration, absolute bias was calculated as the absolute value of the difference between observed beta coefficient and the ‘true’ coefficient across the 10,000 most variable genes in the expression dataset, maximum bias represents maximum absolute bias across these genes

*Average Max* mean of maximum absolute bias across all simulation iterations; *RMS* root mean square of maximum absolute bias value across all simulation iterations

The emboldened values represent the smallest, lowest bias, values under each experimental condition

### Bias after batch correction

Most experimental pipelines recommend batch effect adjustment prior to final analysis. We evaluated two batch adjustment methods, ComBat and a regression adjustment technique. Following batch correction, average maximum absolute bias for ${\upbeta }_{1}$ was similar across all methods but lowest in the optimal condition following regression adjustment for all beta parameters (Table 3). RMS of maximum absolute bias was also lower for ${\upbeta }_{1}$ under the optimal allocation scenario following regression adjustment compared to the other randomization methods and batch correction methods (Table 3 and Fig. 1). This finding was also consistent across ${\upbeta }_{2}$ (age) and ${\upbeta }_{3}$ (HbA1c).

**Table 3:** Maximum absolute bias or difference between true* and batch effect conditions following batch adjustment

		Combat adjustment		Regression adjustment	
		Average max	RMS	Average max	RMS
Case (${\upbeta }_{1})$	Randomization versus True	3.16E-01	1.64E-01	2.89E-01	1.35E-01
	Stratified Randomization versus True	2.74E-01	1.18E-01	2.46E-01	9.05E-02
	Optimal versus True	**1.76E-01**	**1.31E-02**	**1.71E-01**	**1.25E-02**
Age (${\upbeta }_{2})$	Randomization versus True	1.16E-02	5.95E-03	1.05E-02	4.99E-03
	Stratified Randomization versus True	1.14E-02	5.86E-03	1.03E-02	4.62E-03
	Optimal versus True	**6.34E-03**	**4.47E-04**	**6.17E-03**	**4.28E-04**
HbA1c (${\upbeta }_{3})$	Randomization versus True	3.00E-01	1.46E-01	2.68E-01	1.15E-01
	Stratified Randomization versus True	3.01E-01	1.46E-01	2.72E-01	1.16E-01
	Optimal versus True	**1.72E-01**	**1.44E-02**	**1.64E-01**	**1.20E-02**

True* condition represents gene expression values from GSE50397 before batch effects were added to the expression sets in each of the 1000 simulation iterations

Within each experimental iteration, absolute bias was calculated as the absolute value of the difference between observed beta coefficient and the ‘true’ coefficient across the 10,000 most variable genes in the expression dataset, maximum bias represents maximum absolute bias across these genes

*Average Max* mean of maximum absolute bias values across all simulation iterations. *RMS* root mean square of maximum absolute bias value across all simulation iterations

The emboldened values represent the smallest, lowest bias, values under each experimental condition

### Bias at CAPN13: alternative hypothesis condition (${{\varvec{\upbeta}}}_{2}\ne 0 \; \mathbf{a}\mathbf{n}\mathbf{d} \; {{\varvec{\upbeta}}}_{3}\ne 0)$

**Fig. 2:**
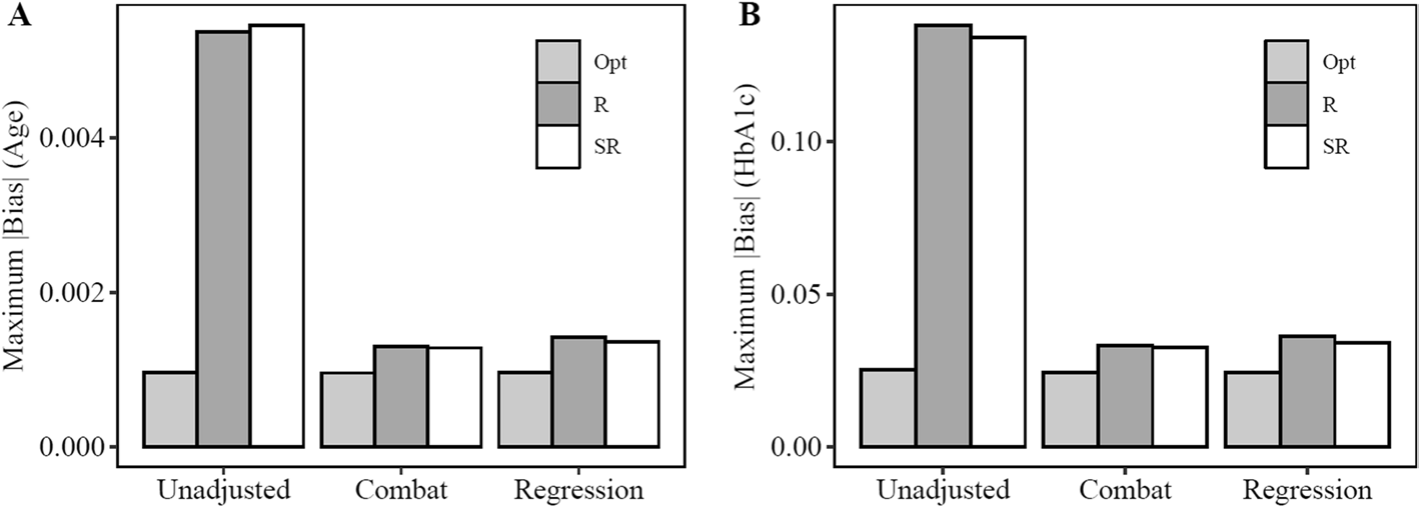
Maximum absolute bias under the alternative hypothesis. In order to understand performance of the optimal allocation algorithm under the alternative hypothesis, bias was estimated at a gene (CAPN13) that was associated with both age and HbA1c in the true dataset. Maximum absolute bias represents the maximum absolute difference between the true betas (prior to adding the batch effects) and the beta estimates under each experimental scenario (Table 1) across all of the 1000 simulation iterations. Maximum absolute bias was estimated for ${\upbeta }_{2}$ (panel A, parameter estimate representing the association between age and gene expression), and ${\upbeta }_{3}$ (panel B, parameter estimate representing the association between HbA1c and gene expression. Opt = optimal allocation strategy, R = simple randomization strategy, SR = stratified randomization strategy

In the results section above, ${\upbeta }_{2}$ and ${\upbeta }_{3}$ are expected to be associated with some but not all genes in the dataset. This is analogous to inclusion of adjustment variables in the statistical modelling framework that are expected to reduce bias in the phenotype gene expression associations, but the associations between these variables and gene expression may not be of interest to the investigator. Therefore, we also evaluated bias at a single gene, CAPN13, that was associated with age and HbA1c and thus, is more likely to be of interest to the investigator. Expression of this gene was used to understand the effectiveness of the optimal allocation method under the alternative hypothesis. Consistent with the primary analysis, mean absolute bias, maximum absolute bias, and root mean square (RMS) of absolute bias were lowest in the optimal allocation group prior to batch correction (Table 4 and Fig. 2). Following batch correction, mean absolute bias, maximum absolute bias, and RMS of absolute bias were consistently lower in the optimal condition relative to the randomization conditions (Table 4 and Fig. 2).

**Table 4:** Absolute bias under alternative hypothesis* in the ‘true’ expression dataset

	No adjustment			ComBat adjustment			Regression adjustment		
	Mean	Max	RMS	Mean	Max	RMS	Mean	Max	RMS
*Age (*${\beta }_{2})$									
Randomization versus True	5.52E-03	5.38E-03	4.05E-02	1.66E-03	1.30E-03	8.59E-03	1.72E-03	1.42E-03	8.54E-03
Stratified Randomization versus True	5.54E-03	5.46E-03	4.07E-02	1.65E-03	1.28E-03	6.26E-03	1.70E-03	1.36E-03	7.73E-03
Optimal versus True	**1.25E-03**	**9.59E-04**	**5.37E-03**	**1.26E-03**	**9.54E-04**	**5.18E-03**	**1.25E-03**	**9.57E-04**	**5.24E-03**
*HbA1c (*${\beta }_{3})$									
Randomization versus True	1.45E-01	1.38E-01	1.04E + 00	4.25E-02	3.30E-02	2.06E-01	4.48E-02	3.61E-02	2.29E-01
Stratified Randomization versus True	1.43E-01	1.34E-01	1.49E + 00	4.25E-02	3.25E-02	1.93E-01	4.26E-02	3.40E-02	1.89E-01
Optimal versus True	**3.27E-02**	**2.51E-02**	**1.28E-01**	**3.21E-02**	**2.42E-02**	**1.30E-01**	**3.20E-02**	**2.43E-02**	**1.31E-01**

^*^Alternative hypothesis based on genes associated with both age and HbA1c in the true expression dataset

*Mean* mean of absolute bias values across all simulation iterations; *RMS* root mean square of absolute bias across all simulation iterations; *Max* max absolute bias value across all simulation iterations; the emboldened values represent the smallest, lowest bias, values under each experimental condition.

### Minimum standard error

**Fig. 3:**
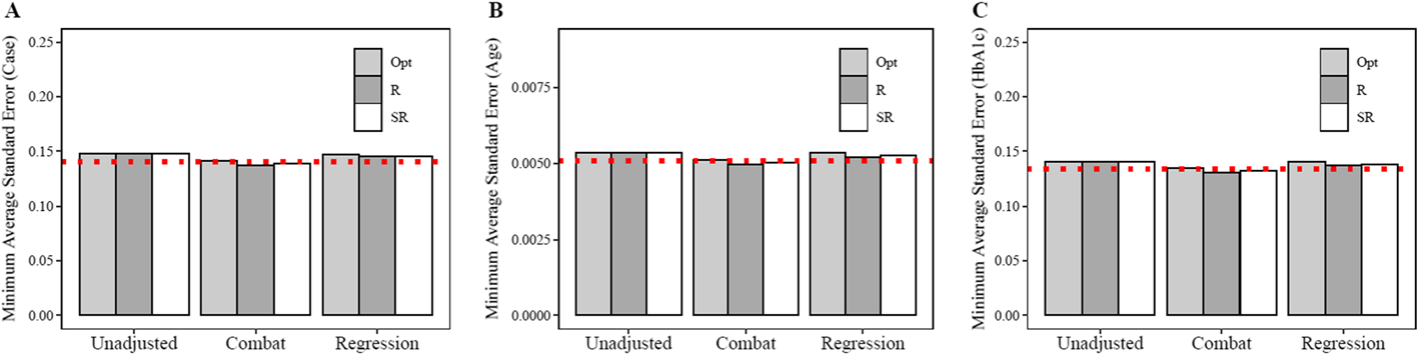
Average minimum standard error of the case parameter estimate. The standard error of the parameter estimates was estimated under each of the experimental scenarios (Table 1) across all 10,000 gene expression features. We estimated the average minimum standard error across all of the 1000 simulation iterations. The average minimum standard error was estimated for ${\upbeta }_{1}$ (panel A, case vs control parameter that was set at 0 to represent the null hypothesis), ${\upbeta }_{2}$ (panel B, parameter estimate representing the association between age and gene expression), and ${\upbeta }_{3}$ (panel C, parameter estimate representing the association between HbA1c and gene expression. Opt = optimal allocation strategy, R = simple randomization strategy, SR = stratified randomization strategy

In addition to minimizing bias, it is desirable to estimate standard errors for the beta parameters that are close to the true error. Overestimation of error increases likelihood of a type II error while underestimation increases the likelihood of a type I error. We evaluated the average of the mean standard error as well as the minimum of the mean standard error across all experimental iterations. Prior to batch correction, standard error estimates for all batch allocation strategies were similar (Table 5 and Fig. 3). After batch correction, standard error estimates decreased in all three batch allocation strategies. Following Combat adjustment, the minimum average standard error was less than the ‘true’ average standard error value for ${\upbeta }_{2}$ (age) and ${\upbeta }_{3}$ (HbA1c) under the randomization and stratified randomization conditions. Using the optimal allocation method, the minimum average standard error was greater than the ‘true’ average standard error value for all parameter estimates before and after batch correction (Table 5 and Fig. 3).

**Table 5:** Standard error before and after batch adjustment

	No adjustment		ComBat adjustment		Regression adjustment	
	Mean	Min	Mean	Min	Mean	Min
Case (${\upbeta }_{1})$ | True Value = 1.40E-01						
Randomization Condition	2.37E-01	1.48E-01	1.42E-01	**1.37E-01**	1.50E-01	1.45E-01
Stratified Randomization Condition	2.38E-01	1.47E-01	1.42E-01	**1.39E-01**	1.49E-01	1.45E-01
Optimal Condition	2.41E-01	1.47E-01	1.42E-01	1.41E-01	1.48E-01	1.47E-01
*Age (*${\beta }_{2})$* | True Value* = *5.09E-03*						
Randomization Condition	8.59E-03	5.35E-03	5.13E-03	**4.97E-03**	5.44E-03	5.21E-03
Stratified Randomization Condition	8.64E-03	5.34E-03	5.13E-03	**5.02E-03**	5.44E-03	5.26E-03
Optimal Condition	8.73E-03	5.35E-03	5.14E-03	5.13E-03	5.35E-03	5.34E-03
*HbA1c (*${\beta }_{3})$* | True Value* = *1.34E-01*						
Randomization Condition	2.26E-01	1.40E-01	1.35E-01	**1.31E-01**	1.43E-01	1.37E-01
Stratified Randomization Condition	2.27E-01	1.40E-01	1.35E-01	**1.32E-01**	1.43E-01	1.38E-01
Optimal Condition	2.29E-01	1.40E-01	1.35E-01	1.35E-01	1.41E-01	1.40E-01

Within each experimental iteration, standard error calculated as the average standard error across the 10,000 most variable genes in the dataset

*Mean* mean of average standard error across all experimental iterations; *Min* minimum of average standard error across all experimental iterations

Red highlighting identifies standard error values less than standard error values estimated in the ‘true’ gene expression dataset (before batch effects were added). The emboldened values represent standard error estimates that are less than the corresponding true value

## Discussion

Batch effects represent an unwanted source of variation in high throughput experiments that have the potential to bias the estimation of biological effects. In the current study, propensity scores were an effective methodology for allocating samples to batches such that all relevant covariates were equally distributed across the batches. The average of maximum absolute bias, as well as the variability of maximum absolute bias across simulations were consistently lower in the optimal, propensity guided allocation strategy compared to alternative randomization strategies.

We compared the effectiveness of our novel batch allocation algorithm relative to simple randomization and stratified randomization batch allocation strategies. In each experimental scenario, prior to batch correction, maximum absolute bias was lower in the optimal allocation conditions compared to the randomization conditions (Table 2). This finding was consistent for all beta estimates representing the null hypothesis (case beta value, β1 = 0). Variability in maximum absolute bias, as represented by the RMS of the maximum bias metrics revealed the largest separation between conditions both pre- and post-batch correction among all probes (See Tables 2, 3 and Fig. 1). This indicates bias is lower and more constrained using the optimal sample allocation strategy. We also evaluated the performance of the algorithm under the alternative hypothesis (β2, association with age) and (β3, association with HbA1c). Not all genes are expected be associated with both age and HbA1c. Therefore, we evaluated the bias in expression of the CAPN13 gene, a gene that was significantly associated with both age and HbA1c levels in the ‘true’ gene expression dataset. Consistent with the null hypothesis results, maximum absolute bias under the alterative hypothesis was consistently lowest in the optimal allocation condition before batch correction (Table 4 and Fig. 2).

The stratified randomization method was the next most effective allocation strategy, outperforming the simple randomization design, especially under the null hypothesis (Tables 3–4 and Fig. 1). In randomized control trials, stratified randomization designs are used to prevent imbalance in the distribution of key confounders across the treatment groups [22]. This is accomplished by independently randomizing treatment assignments within each strata of a key confounder, often sex, age category, site, or clinician. Our study results reinforce the importance of considering stratified randomization designs in high throughput experimental designs.

In cluster randomized trials, covariate constrained randomization provides additional protections against imbalance by considering cluster level factors, such as site location, site volume, etc., during randomization design [21, 23]. However, these designs in RCTs are inherently limited by the prospective nature of enrollment. It is not possible to know all subject level covariates and/or temporally variant cluster level factors prior to treatment assignment. In contrast, in high throughput experiments, it is most cost effective to run samples at one time, after all subject level data has been collected. Therefore, measurable or observed subject level confounders are often known prior to the allocation of samples to batches. The methodology proposed in this study exploits this knowledge by selecting the sample allocation option that minimizes imbalance in confounders across batches. Our approach is conceptually very similar to the OSAT Bioconductor package [24]. However, in contrast to the OSAT package as well as cluster constrained approaches, our approach allows for consideration of multiple continuous variables as well as more complicated interactions between all covariates.

We also evaluated bias after removal of batch effects using the empirical Bayes based ComBat function implemented using the sva R package as well as a linear model regression framework. After adjusting for batch effects, bias estimates from all methods were small, indicating the adjustment methods performed well. Adjustment for batch effects using ComBat had more of an impact on the randomization and stratified randomization conditions due to the higher levels of bias present prior to batch correction. However, a key finding was that the optimal allocation scenario consistently outperformed the other strategies in regard to the mean maximum absolute bias as well as the variability of maximum absolute bias. On average, the randomization methods will produce unbiased estimates. However, running multiple experiments in order to obtain average and thus, unbiased estimates is very costly. The lower variability in maximum absolute bias as well as the consistency in maximum absolute bias before and after batch effect adjustment using our novel optimal allocation method suggests the investigator is less likely to encounter more extreme bias using the optimal allocation approach. This is an important point as batch correction methods do not perform as well in situations where randomization results in an imbalance in key covariates across batches [6].

Under the alternative hypothesis (expression of the CAPN13 gene), the ComBat method slightly outperformed the regression adjustment batch correction method (Table 4) based on RMS of absolute bias. This finding is consistent with Jiao et al., in which the authors concluded that ComBat was the superior method for correcting technical effects including batch and position relative to other batch correction methods evaluated in an analysis of methylation data [25]. In microarray expression data, implementation of ComBat using parametric and non-parametric methods outperformed other commonly used batch correction methods in terms of minimizing proportion of variance attributable to batch effect and improving correlation between technical replicates [1]. ComBat methods were also superior to other techniques according to an overall performance metric, area under the curve, based on sensitivity and specificity of the batch correction methods [1].

Batch adjustment using ComBat has been criticized due to its potential to create artificial associations between high throughput features and biological exposures during the batch correction stage of the analysis [14]. One possible source of bias is due to the underestimation of standard errors. Batch effect correction is performed in two steps and thus, uncertainty in estimating batch effects is not accounted for in the downstream analysis. Therefore, we reviewed minimum average standard errors (in each iteration, standard errors were averaged across all 10,000 features, the minimum average standard error represents the minimum average standard error value across all 1000 of the simulation iterations). As expected, standard errors prior to batch correction were higher in the randomization conditions as well as the optimal randomization condition compared to true biological variation (Table 5). After removal of batch effects, the standard errors for biological exposure (case vs control) as well as the confounders were decreased relative to the unadjusted condition (Table 5 and Fig. 3). Consistent with concerns outlined in previous studies [14, 26], minimum average standard error following ComBat adjustment in the randomization and stratified randomization conditions were less than true average standard error values for the case (${\upbeta }_{1})$, age (${\upbeta }_{2})$, and HbA1c (${\upbeta }_{3})$ parameters (Table 5 and Fig. 3). In contrast, the minimum average standard error values for all parameters estimated using the optimal allocation method were greater than the true standard errors after ComBat adjustment.

### Limitations

The current simulation study has several potential limitations. Performance of our optimal allocation algorithm was based on a single experiment available in the GEO data repository, GSE50397. Parameters for the simulation were based on an array platform. The effectiveness of optimal batch allocation methods under different distributions as well as different normalization strategies should be confirmed in subsequent studies. We only considered one source of batch effects. In high throughput experiments, batch effects are possible during numerous stages of the experiment [2]. We used differences in average propensity scores as our balancing metric. More sophisticated balancing metrics may achieve superior results, especially when there is substantial heterogeneity in propensity score variability across batches. Our simulation framework also assumed no missing covariate data and furthermore, that all covariates are known and measured (no unmeasured confounding). In practice, as with all observational study designs, these assumptions should be rigorously evaluated by the investigator before and after batch correction. Finally, our study assumes that batch effects are known and a balanced group design is achievable (balance in covariate distribution across batches). We acknowledge there are situations where this may not be feasible due to identification of batch effects that were not anticipated during the initial experimental design. Inclusion of technical replicates is a powerful tool to aid in diagnosing and managing these unanticipated batch effects. Batch effects in the context of an unbalanced design is beyond scope of current study. We refer the interested reader to Li et al. [6] for an elegant review of the impact of unbalanced design on batch correction and a potential solution for addressing this concern.

Overall, we developed and tested our sample allocation algorithm under an idealized scenario. The algorithm was based on a relatively small number of samples. We acknowledge that there are situations in which the algorithm may not be computationally feasible. In these more complicated situations, we recommend an adaptation of our algorithm that reviews a computationally feasible number of randomization iterations, based on user input, and selects the randomization iteration that minimizes imbalance in key covariates identified by the investigator across batches. This function is available at https://github.com/carryp/PS-Batch-Effect. We also created a detailed vignette to accompany the functions that describes in detail how to use the functions as well as additional applications of the functions to situations such as random sampling in which balance in key covariates is desirable.

## Conclusions

The current study reinforces the importance of proper experimental design for high throughput studies. Average of maximum absolute bias and variability of maximum absolute bias were consistently minimized in the optimal allocation method. All three allocation strategies performed well following adjustment for batch effects. However, in contrast to the randomization conditions, levels of bias in the optimal allocation condition prior to batch correction were similar to levels of bias after batch correction, indicating the optimal allocation method sets up a robust downstream analysis that minimizes the need for subsequent batch correction. This is an important finding because although batch correction performs well, on average, randomization has the potential to produce sample allocations where covariates are not equally distributed between batches and consequently, batch correction methods do not perform as well. Our optimal sample allocation strategy avoids this scenario by selecting the sample allocation strategy that minimizes imbalance in covariates. As indicated by the lower variability in bias, this strategy is expected to represent a more consistent method for minimize bias. Furthermore, standard error values estimated using the optimal method were never less than the true standard error values. Building on covariate constrained randomization methods [21], our sample allocation methodology allows the investigator to exploit knowledge of subject level confounders to optimize experimental design. Relative to other methods, our algorithm provides additional flexibility to handle continuous variables as well as interactions between covariates. We used the average propensity score as our balancing metric, selecting the sample allocation iteration that minimized differences in average propensity score between batches. Although we focused on an expression set representing array data, we expect the results to generalize to other -omics datasets where features have been transformed to approximate a normal distribution.

## Methods

### Gene expression dataset

We illustrate our novel batch allocation algorithm (Additional file 1) in a hypothetical case–control study. In order to create a biologically plausible scenario, we downloaded a publicly available microarray gene expression dataset from NCBI GEO, GSE50397. The dataset includes gene expression data from 89 human pancreas islet donors. The samples were obtained from Nordic Islet Transplantation Programme, Uppsala University, see http://www.nordicislets.org for more information about islet processing and isolation. Data processing is described in greater detail in previous publications [27–29]. In brief, microarray profiling was performed using the Affymetrix GeneChip^®^ Human Gene 1.0 ST whole transcript platform. Using the oligo R package, the Robust Multi-array Analysis (RMA) method was used to summarize and normalize the array data. Batch correction was performed using COMBAT function from SVA package [7]. The top 10,000 most variable genes from the batch corrected dataset (downloaded from GEO) were used for all subsequent analyses as the ‘true’ gene expression values.

### Phenotype selection

Using the phenotype information available from GSE50397, we selected two variables known to be important in pancreas islet cell gene expression: (1) age and (2) hemoglobin A1c (HbA1c). We focused on individuals with complete age and HbA1c data (*n* = 65). In order to create a scenario where these variables were differentially distributed across the case and control groups, we used a logistic regression model to assign subjects a probability of selection. First, age and BMI were normalized into Z scores so that age and BMI contributed equally to probability of selection. Next, subjects were assigned a probability (P) based on the following: ${\mathrm{P}}_{\mathrm{i}}$ = exp(2.5 + ln(1.5)*${\mathrm{Z Age}}_{\mathrm{i}}$+ ln(1.5)*${\mathrm{Z HbA}1\mathrm{c}}_{\mathrm{i}}$) / 1 + exp(2.5 + ln(1.5)*${\mathrm{Z Age}}_{\mathrm{i}}$+ ln(1.5)*${\mathrm{Z HbA}1\mathrm{c}}_{\mathrm{i}}$). Subjects that were assigned a higher probability were more likely to be older (odds ratio per 1 SD increase in age: 1.5) and have higher HbA1c levels (odds ratio per 1 sd increase in HbA1c level: 1.5). Subjects were then ranked 1 through 65 based on this probability. We then selected the bottom 60 individuals (ranks 6–65) for the study. We preferentially selected lower probabilities to avoid including extreme or outlier age and/or HbA1c values. In this subset of 60 subjects, the bottom 30 were assigned to the case group and the remaining 30 were assigned to the control group. In this model, 2.5 represents the intercept. Given that the subjects were ranked based on the probability of selection (P), the intercept parameter has no influence on sample selection, the same 60 subjects would have been selected and furthermore the same subjects would have been allocated to the case vs control groups regardless of the intercept value.

### Batch assignment

We assigned subjects to batches using three strategies: simple randomization, stratified randomization, and optimal allocation. The simple randomization strategy was implemented by randomly sampling, without replacement, all of the batch labels using the sample() function in r. The stratified randomization used a similar strategy; however, batches were randomly assigned within the case and control strata in order to preserve a balanced design (equal number of cases and controls within each batch). Stratified randomization was executed using the experiment R [30] package (v1.2.0).

**Fig. 4:**
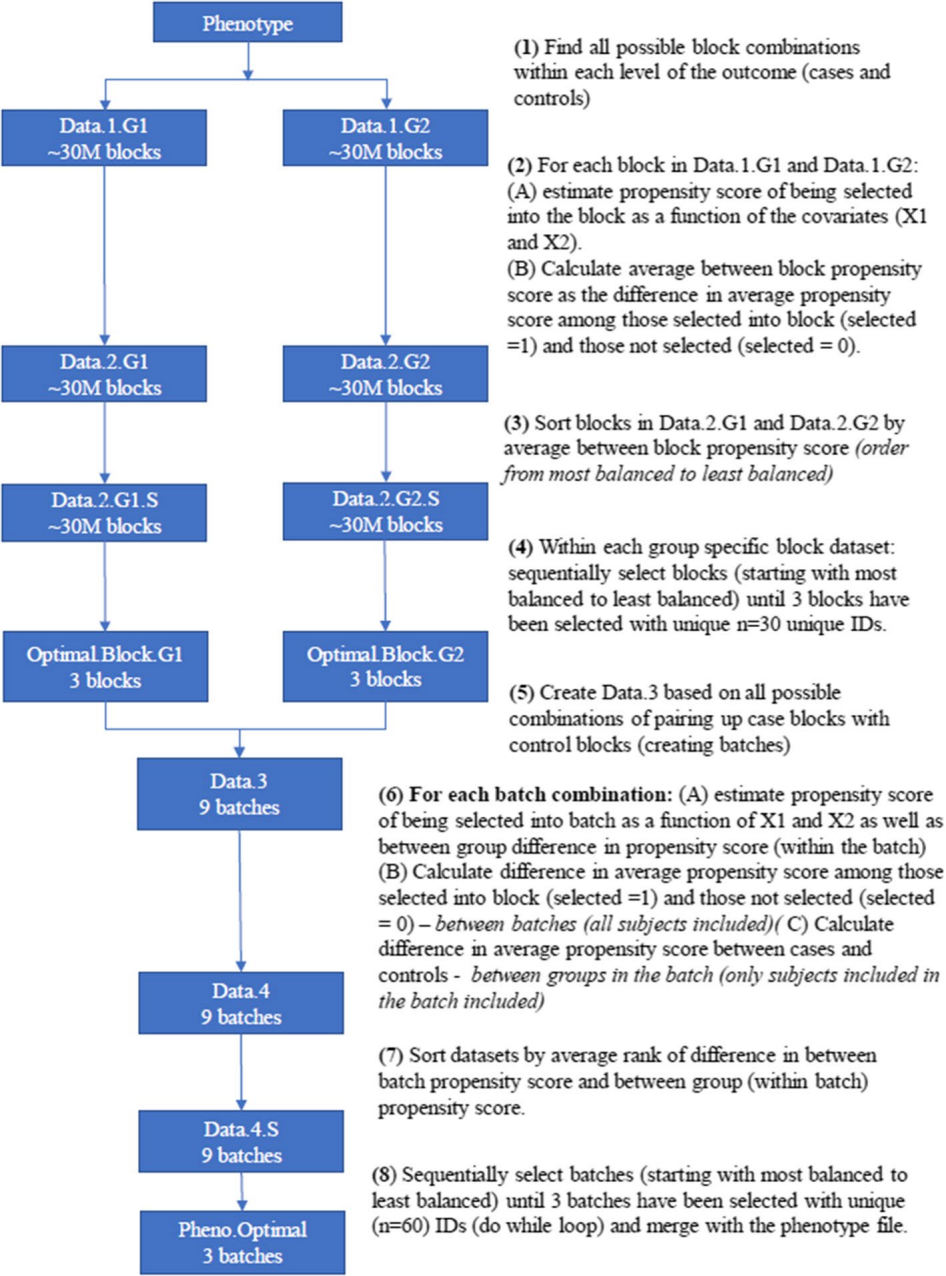
Batch allocation algorithm based on propensity scores. Figure describes the algorithm used to assign subjects to batches. Using a balanced design, the algorithm uses propensity scores in two steps to minimize imbalance in confounders across the study batches

**Fig. 5:**
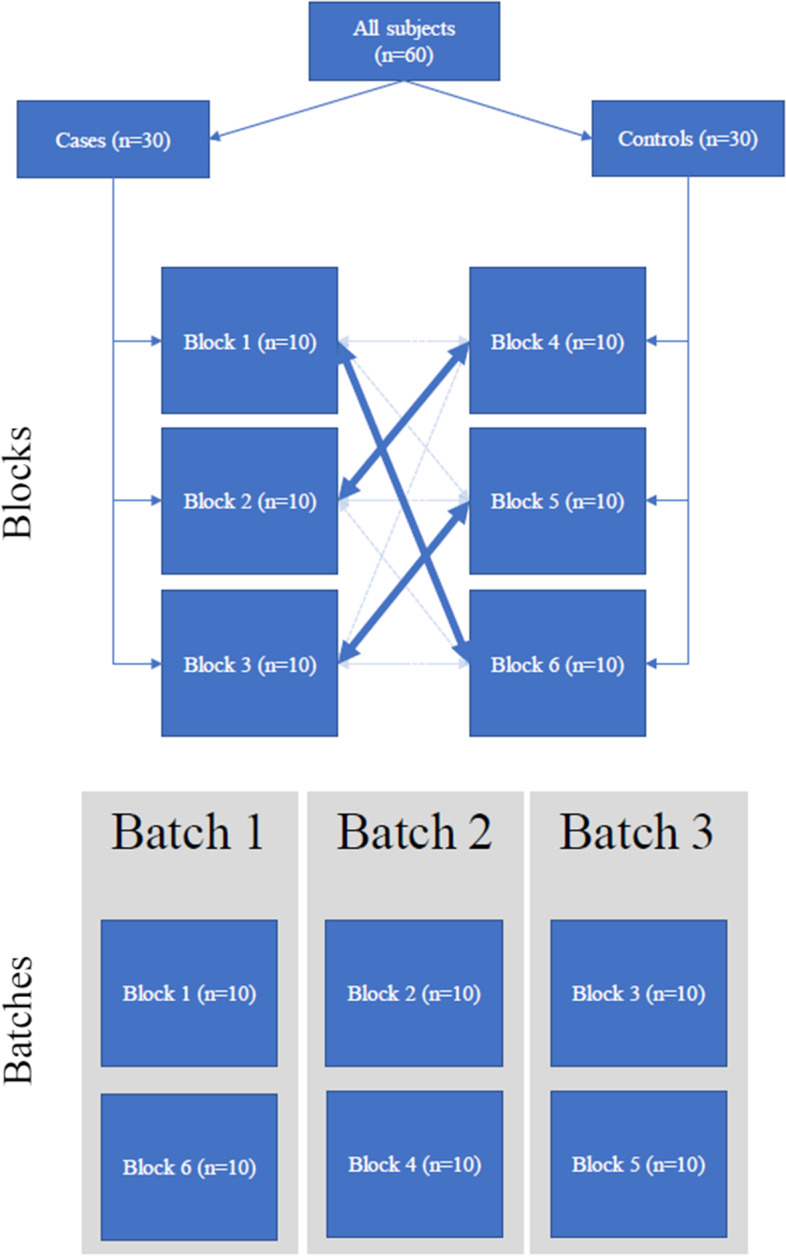
Selection of blocks and batches. Figure provides a visual representation of the batch selection algorithm, highlighting the distinction between blocks and batches. All subjects were split into a case and a control population. All possible blocks of 10 subjects within each population were evaluated and the top 3 blocks were considered. Next, we evaluated all ways of pairing the top 3 case blocks with the top 3 control blocks in order to form the three non-overlapping batches

For the optimal allocation strategy, we used our novel propensity score allocation algorithm (See data availability section, Additional file 1) that considers all possible ways of assigning samples to batches and then selects the allocation that minimizes the difference in average between batch propensity score, see Figs. 4 and 5. In step 1, to maintain a balanced design, 10 subjects from each group (case or control) are assigned to each batch. This creates 3 blocks of 10 subjects per group. We reviewed all possible block combinations. The blocking steps were performed separately for cases and controls to minimize the computational burden. In step 2, once all block combinations were determined, within the case strata, we reviewed the probability (propensity score) of being assigned to each individual block as a function of the covariates, pr(block selection = 1|age, HbA1c). Propensity scores for each block were estimated using a logistic regression model in which batch assignment was the outcome (1 vs 0) and age and HbA1c were included as covariates. For each block, we calculated the difference in average propensity score among subjects assigned to the block versus the average propensity score among all other case subjects not assigned to the block. In step 3, we then sorted all possible blocks by this difference in propensity score. Using this approach, it is possible for the same subject to be present in multiple blocks. Therefore, in step 4, we iteratively selected blocks, starting with the lowest propensity score difference, until 3 blocks of unique subjects had been selected. The same process was repeated in the control strata.

In steps 5 and 6, we considered all possible ways of assigning the three case blocks and the three control blocks to the three batches (see Fig. 5. For each batch combination (one case block and one control block together), we calculated the probability of being selected into the batch using a logistic model, pr(batch selection = 1|Age, HbA1c). Case status was not considered in this step because we used a balanced design (See Fig. 5). We calculated between batch and between group (within a batch) differences in propensity scores. Between batch propensity scores for each batch combination were calculated as the average propensity score among all subjects assigned to the batch compared to the average propensity score among all other subjects. The between group propensity score was calculated as the difference in average propensity score among cases within the selected batch and the average propensity score among controls within the selected batch. The within batch propensity score difference (case vs control in a given batch) and the between group propensity score (selected in batch vs not selected into batch) differences were assigned a rank. In step 7, we then sorted the batch combinations by the average of the between batch propensity score rank and the between group batch propensity score rank. In the final step, we sequentially selected batch combinations starting with the batch combination having the lowest average rank, until 3 batches of unique subjects had been selected. In summary, the propensity scores represent probability of selection conditional on all covariates included in model, providing one metric that captures balance in the distribution of all covariates. Therefore, by selecting the allocation iteration that minimizes (i) the difference in average propensity between batches and, (ii) within a given batch, the iteration that minimizes average propensity scores between cases and controls, we are able to identify the allocation that achieves balance in all covariates.

### Batch effect simulation

In order to simulate batch effects, we used the top 10,000 most variable genes from the gene expression dataset. This dataset represented the ‘true’ gene expression values. We elected to focus on genes with a higher underlying variance based on the assumption that higher variance features in array designs are, in general, are more reproducible [31].

**Fig. 6:**
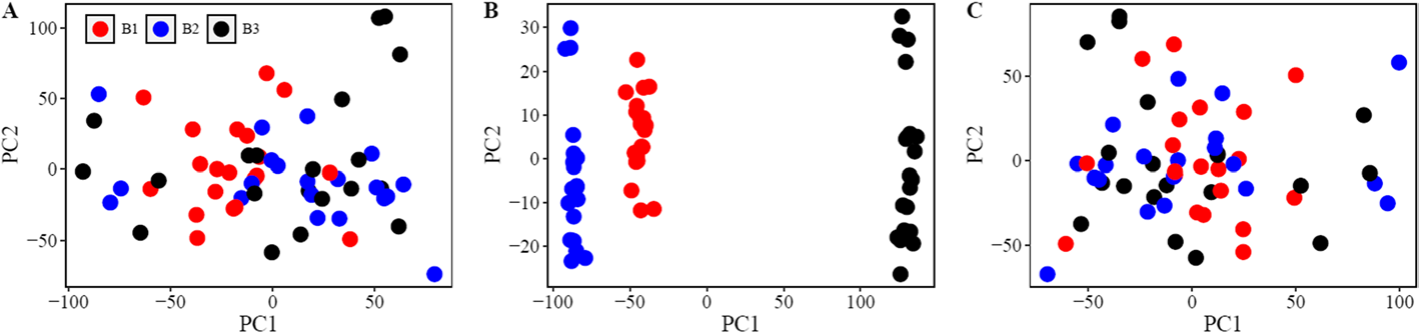
Example of batch effect simulation. A principal component analysis was used to visualize the addition of batch effects to the ‘true expression’ dataset. Each dot represents a subject. In each plot, the y-axis represents PC2 and the x-axis represents PC1 from the principal component analysis of all 10,000 genes included in the microarray gene expression dataset. The panel illustrates the distribution of batches across samples (random) prior to adding batch effects (**A**), after adding batch effects (**B**), and again after batch effects were removed using Combat (**C**)

For each allocation strategy (Table 1), batch effects were simulated by adding a random normal variable to the ‘true’ gene expression values. In each iteration the batch effect was simulated for the three batches using random normal variables, ${\upmu }_{1} \sim \left(0, 0.57\right)$, ${\upmu }_{2} \sim \left(0, 0.57\right),$ and ${\upmu }_{3} \sim \left(0, 0.57\right)$. The variability was set to be approximately twice the median biological variability in the ‘true’ gene expression set. The batch effects were then added to the ‘true’ expression values in addition to a random variable (noise), using ${\mathrm{Y}}_{\mathrm{s}}= {\mathrm{Y}}_{\mathrm{T}}+{\upmu }_{1}*{\mathrm{B}1}_{\mathrm{S}}+ {\upmu }_{2}*{\mathrm{B}2}_{\mathrm{S}}+ {\upmu }_{3}*{\mathrm{B}3}_{\mathrm{S}}+\upomega$, where ${\mathrm{Y}}_{\mathrm{T}}$ is a *n* × 10,000 matrix representing true gene expression values, B1, B2, and B3 are vectors of length *n* of indicator variables (0 or 1) indicating whether subjects are vs are not assigned to batches 1,2, or 3 under each of the scenarios, and ${\upomega }_{\mathrm{s}}$ is an *n* × 10,000 matrix of random error ~ N(0, 0.05) under each of the experimental scenarios (S = optimal allocation, simple random allocation, or stratified randomization allocation scenario). The random error was simulated to be small, less than 1/5 of biological error. Next, we considered two strategies for accounting for batch effects: ComBat, implemented through the sva R package [7] (v3.36.0), and a linear regression approach where batches were adjusted for as fixed effects in a linear regression modelling framework. Using principal component analysis of the 10,000 gene expression features included in the microarray gene expression dataset, Fig. 6 provides a visual representation of batch effects before and after batch effect adjustment under the randomization condition.

### Outcome measures

We considered 9 experimental scenarios to assess the differences in the allocation methods before and after downstream adjustment (see Table 1). Under each experimental scenario, linear regression models were used to estimate the beta parameters for the case variable (${\upbeta }_{1}$), as well as the two confounders $({\upbeta }_{2}\mathrm{ for age and }{\upbeta }_{3}\mathrm{ for HbA}1\mathrm{c},)$. Separate models were fit for each of the genes in the expression dataset. Absolute bias was calculated as the absolute difference between the parameter estimates in each experimental scenario relative to the ‘true’ parameter estimates, ${\mathrm{absolute bias}}_{\mathrm{ie}}$ = ${|\upbeta }_{\mathrm{ie}}^{*}-{\upbeta }_{\mathrm{ie}}|$ where *represents the ‘true’ condition, i represents gene feature within the expression set (i = 1 to 10,000 features) and e represents simulation iteration number (e = 1 to 1,000 iterations). We also estimated the average standard error for each beta across all of the expression features. Next, for each simulation iteration, we calculated, (1) average maximum absolute bias (mean of the maximum absolute bias across all iterations), (2) root mean square (RMS) of absolute maximum bias (standard deviation of the maximum absolute bias across all iterations), (3) mean standard error (mean of the average standard error estimates across all iterations), and (4) minimum average standard error (minimum of the average standard error estimates across all iterations).

We also identified a single transcript that was significantly related to both age and HbA1c levels in the true gene expression dataset. This transcript represented expression of the CAPN13 gene, a protein coding gene that maps to chromosome 2, location 30,945,637 to 31,030,311. We looked at mean absolute bias, maximum absolute bias, and RMS of absolute bias at this gene across all the experimental iterations. Expression of this gene was used to evaluate the performance of our optimal allocation strategy under the alternative hypothesis. The simulation experiment was repeated using larger batch effects (3x and 4x biological variation). The results of these additional simulation experiments are consistent with the primary results and thus, are included as a supplementary files only (see “Additional file 2”).

## Supplementary Information

**Additional file 1.**  This supplementary file includes the code used to develop the optimal allocation algorithm described in the methods section. The following programs are included in this file: (A) data processing, (B) testing association with gene expression, (C) propensity score based sample allocation algorithm, (D) simulating batch effects and testing performance, (E) summarizing performance.

**Additional file 2.** Results of the simulation experiment under different batch effects.

## Data Availability

Source coding for the algorithm included in the Additional file 2. Microarray gene expression data and complete phenotype data are available for download through NCBI GEO, GSE50397. Datasets used in the simulations also included in the Additional file 2. We have also developed a more computationally feasible version of our algorithm to assist in experimental design, the code and accompanying vignette are available here: https://github.com/carryp/PS-Batch-Effect
