# Supplementary material for: Propensity scores as a novel method to guide sample allocation and minimize batch effects during the design of high throughput experiments
Source: BMC Bioinformatics. 2023 Mar 7;24:86. doi: 10.1186/s12859-023-05202-6 (PMC9990331; doi:10.1186/s12859-023-05202-6)
Supplement: Supplementary file 2 — Additional file 2. Results of the simulation experiment under different batch effects. [file 12859_2023_5202_MOESM2_ESM.doc]

| **Appendix Table 1.** Average maximum absolute bias or difference between true* and batch effect conditions prior to batch adjustment | | | | | |
| --- | --- | --- | --- | --- | --- |
|  | | **3x Biological Variation** | | **4x Biological Variation** | |
|  |  | **Average Max** | **RMS** | **Average Max** | **RMS** |
| **Case (**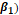 | Randomization vs True | 3.84E-01 | 2.23E-01 | 4.55E-01 | 2.97E-01 |
|  | Stratified Randomization vs True | 3.42E-01 | 1.73E-01 | 3.96E-01 | 2.30E-01 |
|  | Optimal vs True | **1.73E-01** | **1.31E-02** | **1.73E-01** | **1.31E-02** |
| **Age (**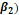 | Randomization vs True | 1.37E-02 | 7.38E-03 | 1.60E-02 | 1.02E-02 |
|  | Stratified Randomization vs True | 1.37E-02 | 7.60E-03 | 1.67E-02 | 1.05E-02 |
|  | Optimal vs True | **6.18E-03** | **4.46E-04** | **6.24E-03** | **4.79E-04** |
| **HbA1c (**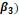 | Randomization vs True | 3.67E-01 | 2.06E-01 | 4.29E-01 | 2.68E-01 |
|  | Stratified Randomization vs True | 3.79E-01 | 2.18E-01 | 4.28E-01 | 2.72E-01 |
|  | Optimal vs True | **1.67E-01** | **1.32E-02** | **1.69E-01** | **1.31E-02** |
| True* condition represents gene expression values from GSE50397 before batch effects were added to the expression sets in each of the 1000 simulation iterations; Within each experimental iteration, absolute bias was calculated as the absolute value of the difference between observed beta coefficient and the ‘true’ coefficient across the 10,000 most variable genes in the expression dataset, maximum bias represents maximum absolute bias across these genes; Average Max = mean of maximum absolute bias across all simulation iterations; RMS = root mean square of maximum absolute bias value across all simulation iterations. The emboldened values represent the smallest, lowest bias, values under each experimental condition. | | | | | |

| **Appendix Table 2.** Maximum absolute bias or difference between true* and batch effect conditions following batch adjustment | | | | | | | | | |
| --- | --- | --- | --- | --- | --- | --- | --- | --- | --- |
|  |  | **3x Biological Variation** | | | | **4x Biological Variation** | | | |
|  |  | **Combat Adjustment** | | **Regression Adjustment** | | **Combat Adjustment** | | **Regression Adjustment** | |
|  |  | **Average Max** | **RMS** | **Average Max** | **RMS** | **Average Max** | **RMS** | **Average Max** | **RMS** |
| **Case (**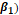 | Randomization vs True | 3.15E-01 | 1.57E-01 | 2.82E-01 | 1.28E-01 | 3.22E-01 | 1.58E-01 | 2.85E-01 | 1.24E-01 |
|  | Stratified Randomization vs True | 2.82E-01 | 1.23E-01 | 2.50E-01 | 1.02E-01 | 2.81E-01 | 1.24E-01 | 2.49E-01 | 9.45E-02 |
|  | Optimal vs True | **1.76E-01** | **1.39E-02** | **1.71E-01** | **1.28E-02** | **1.76E-01** | **1.38E-02** | **1.71E-01** | **1.25E-02** |
| **Age (**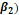 | Randomization vs True | 1.13E-02 | 5.55E-03 | 1.02E-02 | 4.65E-03 | 1.14E-02 | 5.77E-03 | 1.01E-02 | 4.53E-03 |
|  | Stratified Randomization vs True | 1.17E-02 | 5.89E-03 | 1.06E-02 | 5.18E-03 | 1.17E-02 | 5.72E-03 | 1.06E-02 | 4.80E-03 |
|  | Optimal vs True | **6.30E-03** | **4.60E-04** | **6.14E-03** | **4.44E-04** | **6.37E-03** | **4.93E-04** | **6.19E-03** | **4.75E-04** |
| **HbA1c (**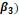 | Randomization vs True | 3.08E-01 | 1.60E-01 | 2.71E-01 | 1.23E-01 | 3.01E-01 | 1.47E-01 | 2.71E-01 | 1.24E-01 |
|  | Stratified Randomization vs True | 3.15E-01 | 1.62E-01 | 2.76E-01 | 1.27E-01 | 3.06E-01 | 1.48E-01 | 2.70E-01 | 1.22E-01 |
|  | Optimal vs True | **1.71E-01** | **1.40E-02** | **1.63E-01** | **1.18E-02** | **1.71E-01** | **1.33E-02** | **1.63E-01** | **1.15E-02** |
| True* condition represents gene expression values from GSE50397 before batch effects were added to the expression sets in each of the 1000 simulation iterations. Within each experimental iteration, absolute bias was calculated as the absolute value of the difference between observed beta coefficient and the ‘true’ coefficient across the 10,000 most variable genes in the expression dataset, maximum bias represents maximum absolute bias across these genes; Average Max = mean of maximum absolute bias values across all simulation iterations. RMS = root mean square of maximum absolute bias value across all simulation iterations. The emboldened values represent the smallest, lowest bias, values under each experimental condition. | | | | | | | | | |

| **Appendix Table 3a.** Absolute bias under alternative hypothesis* in the ‘true’ expression dataset – 3x Biological Variation | | | | | | | | | | |
| --- | --- | --- | --- | --- | --- | --- | --- | --- | --- | --- |
|  |  | **No Adjustment** | | | **ComBat Adjustment** | | | **Regression Adjustment** | | |
|  |  | **Mean** | **Max** | **RMS** | **Mean** | **Max** | **RMS** | **Mean** | **Max** | **RMS** |
| **Age (**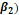 | |  |  |  |  |  |  |  |  |  |
|  | Randomization vs True | 7.97E-03 | 7.35E-03 | 4.79E-02 | 1.68E-03 | 1.30E-03 | 6.78E-03 | 1.66E-03 | 1.32E-03 | 6.80E-03 |
|  | Stratified Randomization vs True | 8.06E-03 | 7.72E-03 | 7.82E-02 | 1.69E-03 | 1.27E-03 | 7.34E-03 | 1.78E-03 | 1.39E-03 | 7.61E-03 |
|  | Optimal vs True | 1.24E-03 | 9.40E-04 | 5.05E-03 | 1.24E-03 | 9.29E-04 | 5.27E-03 | 1.24E-03 | 9.36E-04 | 5.32E-03 |
| **HbA1c (**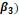 | |  |  |  |  |  |  |  |  |  |
|  | Randomization vs True | 2.15E-01 | 2.06E-01 | 1.30E+00 | 4.45E-02 | 3.51E-02 | 2.46E-01 | 4.39E-02 | 3.75E-02 | 2.60E-01 |
|  | Stratified Randomization vs True | 2.28E-01 | 2.18E-01 | 1.61E+00 | 4.46E-02 | 3.49E-02 | 2.20E-01 | 4.43E-02 | 3.55E-02 | 3.16E-01 |
|  | Optimal vs True | 3.19E-02 | 2.50E-02 | 1.36E-01 | 3.09E-02 | 2.44E-02 | 1.25E-01 | 3.02E-02 | 2.43E-02 | 1.24E-01 |
| *Alternative hypothesis based on genes associated with both age and HbA1c in the true expression dataset; Mean = mean of absolute bias values across all simulation iterations; RMS = root mean square of absolute bias across all simulation iterations; Max = max absolute bias value across all simulation iterations. | | | | | | | | | | |

| **Appendix Table 3b.** Absolute bias under alternative hypothesis* in the ‘true’ expression dataset – 4x Biological Variation | | | | | | | | | | |
| --- | --- | --- | --- | --- | --- | --- | --- | --- | --- | --- |
|  |  | **No Adjustment** | | | **ComBat Adjustment** | | | **Regression Adjustment** | | |
|  |  | **Mean** | **Max** | **RMS** | **Mean** | **Max** | **RMS** | **Mean** | **Max** | **RMS** |
| **Age (**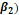 | |  |  |  |  |  |  |  |  |  |
|  | Randomization vs True | 4.73E-02 | 3.74E-02 | 2.24E-01 | 1.62E-03 | 1.31E-03 | 8.80E-03 | 4.43E-02 | 3.56E-02 | 2.45E-01 |
|  | Stratified Randomization vs True | 4.34E-02 | 3.41E-02 | 1.92E-01 | 1.66E-03 | 1.34E-03 | 7.49E-03 | 4.27E-02 | 3.59E-02 | 2.66E-01 |
|  | Optimal vs True | 3.27E-02 | 2.46E-02 | 1.49E-01 | 1.17E-03 | 8.98E-04 | 5.53E-03 | 3.18E-02 | 2.41E-02 | 1.64E-01 |
| **HbA1c (**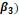 | |  |  |  |  |  |  |  |  |  |
|  | Randomization vs True | 4.66E-02 | 3.87E-02 | 2.58E-01 | 1.60E-03 | 1.33E-03 | 8.40E-03 | 4.47E-02 | 3.69E-02 | 2.62E-01 |
|  | Stratified Randomization vs True | 4.28E-02 | 3.28E-02 | 1.99E-01 | 1.70E-03 | 1.38E-03 | 9.47E-03 | 4.26E-02 | 3.44E-02 | 2.43E-01 |
|  | Optimal vs True | 3.27E-02 | 2.46E-02 | 1.50E-01 | 1.17E-03 | 8.93E-04 | 5.28E-03 | 3.17E-02 | 2.39E-02 | 1.54E-01 |
| *Alternative hypothesis based on genes associated with both age and HbA1c in the true expression dataset; Mean = mean of absolute bias values across all simulation iterations; RMS = root mean square of absolute bias across all simulation iterations; Max = max absolute bias value across all simulation iterations. | | | | | | | | | | |

| **Appendix Table 4a.** Standard error– 3x Biological Variation | | | | | | | |
| --- | --- | --- | --- | --- | --- | --- | --- |
|  |  | **No Adjustment** | | **ComBat Adjustment** | | **Regression Adjustment** | |
|  |  | **Mean** | **Min** | **Mean** | **Min** | **Mean** | **Min** |
| **Case (**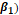 **| True Value = 1.40E-01** | |  |  |  |  |  |  |
|  | Randomization Condition | 3.19E-01 | 1.48E-01 | 1.41E-01 | 1.37E-01 | 1.50E-01 | 1.45E-01 |
|  | Stratified Randomization Condition | 3.21E-01 | 1.48E-01 | 1.41E-01 | 1.39E-01 | 1.49E-01 | 1.46E-01 |
|  | Optimal Condition | 3.25E-01 | 1.48E-01 | 1.42E-01 | 1.41E-01 | 1.48E-01 | 1.47E-01 |
| **Age (**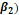 **| True Value = 5.09E-03** | |  |  |  |  |  |  |
|  | Randomization Condition | 1.16E-02 | 5.36E-03 | 5.13E-03 | 4.98E-03 | 5.43E-03 | 5.25E-03 |
|  | Stratified Randomization Condition | 1.16E-02 | 5.35E-03 | 5.13E-03 | 5.03E-03 | 5.44E-03 | 5.29E-03 |
|  | Optimal Condition | 1.18E-02 | 5.35E-03 | 5.14E-03 | 5.12E-03 | 5.35E-03 | 5.34E-03 |
| **HbA1c (**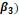 **| True Value = 1.34E-01** | |  |  |  |  |  |  |
|  | Randomization Condition | 3.04E-01 | 1.41E-01 | 1.35E-01 | 1.31E-01 | 1.43E-01 | 1.38E-01 |
|  | Stratified Randomization Condition | 3.05E-01 | 1.41E-01 | 1.35E-01 | 1.32E-01 | 1.43E-01 | 1.38E-01 |
|  | Optimal Condition | 3.10E-01 | 1.41E-01 | 1.35E-01 | 1.35E-01 | 1.41E-01 | 1.40E-01 |
| Mean = mean of average standard error across all experimental iterations; Min = minimum of average standard error across all experimental iterations; Red highlighting identifies standard error values less than standard error values estimated in the ‘true’ gene expression dataset (before batch effects were added) | | | | | | | |

| **Appendix Table 4b.** Standard error– 4x Biological Variation | | | | | | | |
| --- | --- | --- | --- | --- | --- | --- | --- |
|  |  | **No Adjustment** | | **ComBat Adjustment** | | **Regression Adjustment** | |
|  |  | **Mean** | **Min** | **Mean** | **Min** | **Mean** | **Min** |
| **Case (**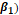 **| True Value = 1.40E-01** | |  |  |  |  |  |  |
|  | Randomization Condition | 3.93E-01 | 1.47E-01 | 1.41E-01 | 1.39E-01 | 1.50E-01 | 1.45E-01 |
|  | Stratified Randomization Condition | 3.96E-01 | 1.47E-01 | 1.41E-01 | 1.38E-01 | 1.49E-01 | 1.45E-01 |
|  | Optimal Condition | 4.02E-01 | 1.47E-01 | 1.42E-01 | 1.41E-01 | 1.48E-01 | 1.47E-01 |
| **Age (**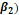 **| True Value = 5.09E-03** | |  |  |  |  |  |  |
|  | Randomization Condition | 1.43E-02 | 5.34E-03 | 5.13E-03 | 5.05E-03 | 5.44E-03 | 5.27E-03 |
|  | Stratified Randomization Condition | 1.44E-02 | 5.34E-03 | 5.13E-03 | 5.01E-03 | 5.44E-03 | 5.26E-03 |
|  | Optimal Condition | 1.46E-02 | 5.34E-03 | 5.13E-03 | 5.12E-03 | 5.35E-03 | 5.34E-03 |
| **HbA1c (**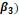 **| True Value = 1.34E-01** | |  |  |  |  |  |  |
|  | Randomization Condition | 3.75E-01 | 1.40E-01 | 1.35E-01 | 1.33E-01 | 1.43E-01 | 1.39E-01 |
|  | Stratified Randomization Condition | 3.77E-01 | 1.40E-01 | 1.35E-01 | 1.32E-01 | 1.43E-01 | 1.38E-01 |
|  | Optimal Condition | 3.83E-01 | 1.40E-01 | 1.35E-01 | 1.35E-01 | 1.41E-01 | 1.40E-01 |
| Mean = mean of average standard error across all experimental iterations; Min = minimum of average standard error across all experimental iterations; Red highlighting identifies standard error values less than standard error values estimated in the ‘true’ gene expression dataset (before batch effects were added) | | | | | | | |
