# Supplementary material for: Propensity scores as a novel method to guide sample allocation and minimize batch effects during the design of high throughput experiments
Source: BMC Bioinformatics. 2023 Mar 7;24:86. doi: 10.1186/s12859-023-05202-6 (PMC9990331; doi:10.1186/s12859-023-05202-6)
Supplement: Supplementary file 1 — Additional file 1. This supplementary file includes the code used to develop the optimal allocation algorithm described in the methods section. The following programs are included in this file: (A) data processing, (B) testing association with gene expression, (C) propensity score based sample allocation algorithm, (D) simulating batch effects and testing performance, (E) summarizing performance. [file 12859_2023_5202_MOESM1_ESM.zip › READ ME.doc]

**Propensity Score Based Algorithm to Optimize Sample Allocation**

The purpose of this repository is to describe the sample allocation algorithm described in the manuscript entitled “Propensity Scores as a Novel Method to Guide Sample Allocation and Minimize Batch Effects During the Design of High Throughput Experiments”. This repository includes programs for (A) data processing, (B) testing association with gene expression, (C) propensity score based sample allocation algorithm, (D) simulating batch effects and testing performance, (E) summarizing performance.

**Summary:**

(A) **Processing -** The 0_Data Processing_GitHub.R program describes initial data processing steps. The microarray gene expression dataset was initially accessed from NCBI GEO (GSE50397). A phenotype ( Pheno.Batch.Effect.Rda) and expression matrix dataset (Expression.Data.Rda) are created in this step, both datasets are used in subsequent steps.

(B) **‘True’ Gene Expression Associations** – The 1_Expression Association_GitHub.R program is used to test the association between gene expression levels in the ‘true’ dataset (prior to adding batch effects) and BMI and age in the phenotype file. Output from this analysis is used to judge the effectiveness of the batch effect correction methods tested in subsequent steps.

(C) **Creating Optimal Allocation Dataset –** The 2_Creating Optimal Permutation Dataset_GitHub.R program outlines algorithm for identifying optimal allocation of samples to batches. The algorithm follows steps described in Figures 4 and 5 in the manuscript.

(D) **Testing Algorithm Performance** - The 3_Simulation Testing_Batch Effect_GitHub.R program outlines strategy for simulating batch effects and testing performance of optimal allocation algorithm relative to randomization and stratified randomization.

(E) **Summarizing Algorithm Performance -** The 4_Batch Effect Simulation Output_GitHub.R program summarizes procedures for summarizing the simulation output dataset (Simulation.Output.Batch.Effect.Revised.Rda) in order to recreate tables described in the manuscript.

All datasets used in the programs are stored in the “Data” folder. The programs describe process for creating these datasets as well as using them to test the effectiveness of the optimal allocation algorithm.
